# The complexity of epigenetic diseases

**DOI:** 10.1002/path.4647

**Published:** 2015-11-17

**Authors:** Ailbhe Jane Brazel, Douglas Vernimmen

**Affiliations:** ^1^ The Roslin Institute, Developmental Biology DivisionUniversity of EdinburghEaster BushMidlothianUK

**Keywords:** enhancer, epigenetics, chromatin, cancer, leukaemia, mutations

## Abstract

Over the past 30 years, a plethora of pathogenic mutations affecting enhancer regions and epigenetic regulators have been identified. Coupled with more recent genome‐wide association studies (GWAS) and epigenome‐wide association studies (EWAS) implicating major roles for regulatory mutations in disease, it is clear that epigenetic mechanisms represent important biomarkers for disease development and perhaps even therapeutic targets. Here, we discuss the diversity of disease‐causing mutations in enhancers and epigenetic regulators, with a particular focus on cancer. © 2015 The Authors. *The Journal of Pathology* published by John Wiley & Sons Ltd on behalf of Pathological Society of Great Britain and Ireland.

## Introduction

The modern polymath Conrad H Waddington (1905–1975) was the first to coin the term ‘epigenetics’ to describe heritable changes in gene expression not caused by changes in the DNA sequence 1. Now, we know that the DNA is segregated into chromosomes inside the nucleus of each cell and that packaging proteins, called histones, associate with DNA to form the chromatin. Nucleosomes are the basic units of the chromatin and are formed by an octamer of histones (two copies of a tetramer; H2A, H2B, H3, and H4). If the DNA is well packed in these nucleosomes (condensed chromatin), genes will be switched off, whereas if the DNA is uncovered, as in decondensed chromatin, and thus more accessible to transcription factors (TFs), genes are more likely to be switched on. The level of compaction of these nucleosomes is influenced by chemical tags or ‘epigenetic modifications’, which associate with the histones or directly with the DNA. Just as each organism has its own unique DNA sequence, each cell type at each developmental stage has a distinctive epigenetic modification profile. As such, cellular commitment and differentiation are by definition an epigenetic phenomenon 2. Critically, the presence of these epigenetic modifications can be associated with changes in the environment (eg diet, stress, smoke inhalation, etc).

Genetic diseases are caused by a variety of mutations affecting the genes or the regulatory regions (promoters, enhancers, etc) controlling the expression of these genes, as well as by chromosomal alteration, such as translocations or aneuploidy. Enhancers are regulatory regions that increase the rate or the probability of transcription of a target gene. An enhancer may lie far away, upstream or downstream from the gene that it regulates or may be located in an intron of its target gene 3, 4. Mutations of enhancer sequences, and of the protein factors regulating enhancer function, contribute to a growing class of ‘enhanceropathies’ 5. α‐ and β‐thalassaemia are key examples of monogenic diseases which can be caused by the deletion of remote enhancers in certain patients 6.

Epigenetic alterations including DNA methylation and histone post‐translational modifications are catalysed by families of epigenetic regulators such as DNA and histone methyltransferases. Only five DNA modifications have been identified in eukaryotes, whereas approximately 130 specific histone modifications have been described, grouped into 16 classes 7, 8, 9. These histone modifications involve many different amino acids on each histone protein and have specific functions 7, 8. Epigenetic modifications can be generated by ‘writer’ enzymes and removed by ‘eraser’ enzymes. Specialized ‘reader’ proteins contain unique domains that specifically recognize these modifications and use them as docking sites 10. Some epigenetic regulators are required for transcriptional regulation, DNA repair, cell cycle, and differentiation – hence their important role in many cancers.

Multigenic diseases (eg cancer) result from the accumulation of mutations in genes such as oncogenes (gain‐of‐function mutations) and/or tumour suppressor genes (loss‐of‐function mutations). This causes a loss of coordination between proliferation and differentiation of progenitor cells. Diseases involving mutations of epigenetic regulators have been recently described in a variety of solid tumours and blood malignancies 8. This has highlighted the importance of epigenetics in disease, but also implies that these diseases are genetic after all.

In this review, we will discuss the complexity of pathogenic mutations and single nucleotide polymorphisms (SNPs) affecting enhancer activity. Next, we will look at the importance of epigenetic signatures that are associated with diseases as biomarkers for disease development. We will then discuss the role that epigenetic regulator mutations play in disease and the interplay of these genetic mutations and pure epigenetic mutations. Although unique research advantages become available when studying different model organisms it is important to note the fundamental differences in the epigenetic regulation between different species, which we will discuss throughout this review.

## The molecular basis of aberrant gene expression

**Figure 1:**
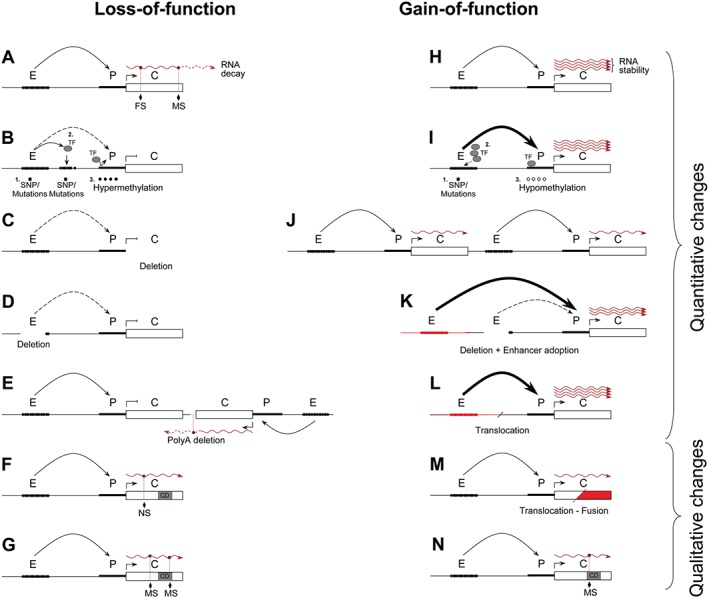
Molecular basis of genetic diseases. Effects of loss‐ and gain‐of‐function mutations affecting gene expression are quantitative and/or qualitative. (A) A missense mutation or a small insertion/deletion mutation (frameshift) in a coding sequence or at a PolyA signal often leads to abortive translation or RNA decay 162. (B) Reduction of chromosomal looping between the enhancer and the promoter might be due to (1) natural variant or mutation at the enhancer 163, (2) the presence of a new SNP forming a new enhancer/promoter region which titrates the remote enhancer activity 43, or (3) promoter or enhancer hypermethylation 164. (C) Deletion of the gene 165. (D) Deletion of the remote enhancer 166. (E) Deletion of the PolyA signal of a downstream and convergent gene, leading to the production of antisense RNA 167. (F) Nonsense mutation adding a new premature stop codon producing a truncated protein 168. Note that truncated proteins may also have a gain‐of‐function activity 169. (G) Missense mutation affecting the non‐enzymatic activity or abolishing the catalytic domain of an enzyme 104. (H) Normal rate of transcription, but increased accumulation of final gene product due to the presence of an RNA 170 or a protein 171 stabilizing molecule. (I) Increased enhancer activity due to (1) enhancer mutation 25, (2) overexpression of a transcription factor 172, or (3) promoter hypomethylation 173. (J) An increase in gene copy number, including regulatory regions 174. (K) Large genomic deletion bringing a strong (but irrelevant) enhancer closer 175. (L) Translocation with a heterologous chromosome (red) creating a fusion locus with a new strong enhancer regulating an illegitimate gene 176. (M) Translocation with a heterologous chromosome (red) producing a fusion gene, with increased biological activity 96. (N) Missense mutation improving enzymatic activity 81. E, enhancer; P, promoter; C, coding region; TF, transcription factor; CD, catalytic domain; MS, missense mutation; NS, nonsense mutation; FS, frameshift mutation. Dashed curved arrows represent impaired enhancer–promoter interaction (looping); thin curved arrows, normal looping; and thick curved arrows, strong looping. Wavy red lines indicate mRNA.

The effects of loss‐ and gain‐of‐function mutations can be quantitative or qualitative. These are summarized in Figure 1. The nature of a disease‐inducing mutation greatly influences the types of tests needed to diagnose and the therapeutic approaches used. A quantitative change directly causes an increase or a decrease in abundance of the final gene product. Quantitative changes in expression are relatively easily detected with classical and low‐cost techniques such as immunohistochemistry (IHC), fluorescence *in situ* hybridization (FISH), colorimetric *in situ* hybridization (CISH), and real‐time quantitative polymerase chain reaction (qPCR) 11. Qualitative changes can alter the function of a mutated gene. Their detection may require more sophisticated and high‐cost sequencing techniques such as targeted exome sequencing or whole genome sequencing (which can now be achieved at a single cell level) 12, 13, 14. However, when a qualitative mutation occurs in a master regulator, this often influences the transcription levels of downstream target genes, which can be measured using quantitative means. For example, acute lymphoblastic leukaemia (ALL) patients with *MLL* rearrangements (discussed below) have a distinct gene expression profile which distinguishes them from other ALL patients and acute myeloid leukaemia (AML) patients 15.

## Transcriptional enhancers and diseases

### Loss‐of‐function enhancer mutations

Thirty years ago, human genetics studies pioneered the identification of functional remote regulatory elements in patients with α‐ and β‐thalassaemia (reviewed in ref 6). In most cases, a deletion removing a *globin* gene causes its down‐regulation (Figure 1C). However, in rare cases, the genes (including their promoters) remain intact but the deletion of one (or several) remote enhancer(s) causes their down‐regulation (Figure 1D). There are many other instances in which enhancer deletions have been shown to cause pathologies. Deletions in enhancers of *FOXL2* 16, *POU3F4* 17, *SOST* 18, 19, and *SOX10* 20, 21 have been linked to blepharophimosis syndrome, X‐linked deafness type 3, van Buchem disease, and Waardenburg syndrome type 4, respectively.

Deletions are not the only mutations affecting enhancer function. Although the exact mechanism of pathogenesis is currently unclear, a variety of SNPs can affect enhancer activity by changing TF binding affinity and/or specificity (Figures 1B and 1I). Hirschsprung disease (HSCR), a multigenic, heritable disorder affecting the ganglion cells in the large intestine or gastrointestinal tract, is an example. Less than 30% of HSCR patients have identified mutations in the coding sequence of candidate genes, such as *RET* (encoding a tyrosine kinase receptor), but SNPs within the enhancers of either the *SOX10* (a transcription factor regulating *RET* expression) or the *RET* genes have been identified in other patients 20, 21, 22. SNPs in *SOX10* enhancers in isolated HSCR and Waardenburg syndrome type 4 patients (a rare condition characterized by deafness and pigmentation anomalies) have been shown to significantly reduce *Sox10* expression, also leading to down‐regulation of *RET* expression 20, 21. A single base‐pair change in one of the *RET* enhancers is also overrepresented in affected populations 22. This SNP reduces the activity of the enhancer in gene reporter assays compared with the normal allele, apparently by disruption of a SOX10 binding site which subsequently reduces *RET* expression 23.

### Gain‐of‐function enhancer mutations

One example of gain‐of‐function pathogenic mutations identified in enhancer sequences is in patients with preaxial polydactyly 24, 25. Point mutations in the long‐distant, limb‐specific enhancer for *sonic hedgehog* (*SHH*) can cause ectopic expression of this gene, leading to the formation of extra digits in human and other animal patients 26.

More recently, high‐throughput sequencing technology has allowed GWAS to identify a large number of candidate SNPs associated with diseases 27, 28. A number of independent GWAS have identified distinct breast, prostate, and colon cancer risk regions in the 8q24 region, each enriched with histone modifications that are characteristic of enhancers 29. Within these enhancer regions, various SNPs have been identified and that predispose susceptibility to certain cancer types. For example, the prostate cancer risk allele rs11986220 exhibits stronger binding to the TF forkhead box protein A1 (FOXA1) 30. This increased binding of FOXA1 can facilitate the recruitment of FOXA1‐dependent androgen receptor, which is associated with poor prognosis in prostate cancer 31.

Using chromatin conformation capture (3C) technology 32, a number of risk regions in the 8q24 region have been shown to form large chromosomal loops to the promoter of the *MYC* oncogene 29, 30, 33, 34, 35, 36. However, none of these studies has successfully demonstrated a correlation between the occurrence of these SNPs and an increase in downstream *MYC* expression. *MYC* expression may be enhanced by these SNPs, but only at specific times during tumourigenesis, or only in a particular subset of cells (eg cancer stem cells). The prostate cancer risk locus at 8q24 also forms contacts with multiple other genomic loci, sometimes in a cell‐type‐specific manner, suggesting that the pathogenic mechanisms of identified susceptibility alleles may be *MYC*‐independent 37. Of note, a number of susceptibility alleles in the 8q24 region have been shown to increase the expression of the oncogene *PVT1* 35.

In cells of the same type but from different individuals, SNPs associated with disease (quantitative trait loci; QTL) affect the variability of TF binding and therefore can lead to changes in the associated chromatin state. This would cause local epigenetic variability between individuals. Recently, Waszak *et al* and Grubert *et al* found that such local chromatin changes due to distinct genetic variation at TF binding sites are also influenced by the state of other regulatory elements (local, but also hundreds of kilobases away), and thus affect large genomic compartments forming regulatory units, called variable chromatin modules (VCMs) 38. Variability within each of these VCMs is mediated by the spatial chromatin interactions 39, which may affect the expression of several genes. This might also suggest that very few apparent ‘epi‐mutations’ might be wholly distinguishable from DNA sequence changes.

## Epigenetic signatures of disease

True epi‐mutations (ie epigenetic modifications differentially represented in a diseased versus healthy population) represent important biomarkers 40, which can be exploited for patient stratification 41, 42, identification of candidate pathways in disease 43, 44, and potential targets for novel epigenetic editing therapies 45. Disease‐specific DNA methylomes have been identified in patients with active ovarian cancer 46, distinct forms of AML 47, colorectal cancer 48, and other diseases. Thousands of loci have been found to be differentially enriched for epigenetic signatures marking enhancers (monomethylation of lysine 4 at histone H3) in a given colorectal cancer cell sample when compared with normal crypt cells 49.

EWAS are now underway to identify epi‐mutations associated with disease 50. These studies are focused mainly on changes of DNA methylation (methylation quantitative trait loci – methQTLs) as this is more feasible than histone modification analyses. EWAS have already identified differentially methylated genomic regions that may mediate the epigenetic risk of rheumatoid arthritis 51 and that may be induced by regular smoking 52, 53. Intrinsic challenges in epigenetic analyses include the epigenetic variance between different cell types and different developmental stages. New study design and analysis techniques are now being developed to help circumvent these issues 54, 55. However, epigenetic variance between single cells of the same type and development stage may also cause difficulty in separating true signals from noise 50, 56.

## Epigenetic regulation in disease

### Regulators of DNA modifications

Five different DNA modifications have been described in eukaryotes. The methylation of number 5 carbon on cytosine residues (5mC) in CpG dinucleotides was the first described covalent modification of DNA 57. 5mC oxidative intermediates such as 5‐hydroxymethylcytosine (5hmC), 5‐formylcytosine (5fC), and 5‐carboxylcytosine (5caC) are other metabolites found at CpGs 8. Recently, a new modification of eukaryote DNA, N6‐methyladenine, was described 9.

In vertebrates, DNA regions with a high density of CpG dinucleotides form CpG islands. These are short (∼1000 bp) interspersed CpG‐rich and predominantly unmethylated DNA sequences 58. They are found in all housekeeping genes and in a proportion of tissue‐specific and developmental regulator genes. Although DNA methylation is well documented in vertebrates, it is less well understood in other organisms. In fact, the most commonly studied invertebrate model organisms, the fly *Drosophila melanogaster* and the worm *Caenorhabditis elegans*, and also the fungus *Saccharomyces cereviceae* all lack DNA methylation 58. However, in some insects, such as the Hymenoptera honey bee (*Apis mellifera*, discussed later), DNA methylation occurs but is primarily found in gene bodies affecting the splicing of ubiquitously expressed genes 59. In mammals, however, DNA methylation appears in intergenic regions, where it can, for example, impede TF binding at promoter regions 58 (Figure 1B).

### DNA modifications and disease

Methylated CpG dinucleotides are more sensitive to mutation by deamination to TpG or CpA 60, and thus represent a key example where epi‐mutations can generate genetic mutations. Early studies found that CpG islands are underrepresented in the rodent compared with the human genome, as they have been eroded during evolution 61, 62. These studies suggest that CpG dinucleotides within the mouse CpG islands were accidentally methylated and mutated to TpG or CpA during evolution. This could have dramatic consequences when studying a mouse model where the gene of interest might be regulated differently compared with its human orthologue. For example, the human *α‐globin* gene is regulated by Polycomb group repressive complexes during differentiation, whereas the mouse *α‐globin* gene is not 6. This led to the development of a humanized mouse model for the *in vivo* study of the regulation of the human *α‐globin* gene expression 63.

Cytosine is methylated by a family of enzymes called *de novo* (*DNMT1*) and maintenance (*DNMT3*) DNA methyltransferases. One of the DNMTs, *DNMT3A*, is inactivated in related haematological malignancies 64 such as myelodysplastic syndromes (MDS) 65 and AML 66. Around 30% of MDS cases progress to acute myeloid leukaemia (AML) 67. Interestingly, loss‐of‐function mutations of *DNMT3A* that do not affect its catalytic domain disrupt the formation of a tetramer with another protein, *DNMT3L* 68, 69. These mutations have a dominant‐negative effect, which prevents the wild‐type protein from functioning normally 68, 69. The ten–eleven translocation (TET 1–3) family of proteins are the mammalian DNA hydroxylases responsible for catalytically converting 5mC to 5hmC 70. Loss‐of‐function of *TET2* and *DNMT3A* seems to be a primary event during leukaemogenesis 71. Disruption of normal methylation patterns in colorectal cancer cells correlates with underexpression of tumour suppressor genes (Figure 1B) and overexpression of oncogenes (Figure 1I) 48.

### Regulators of histone modifications

The opposing effects of the Polycomb group (PcG, associated with gene repression) and Trithorax group (associated with gene activation) remodelling proteins regulate many cellular decisions in stem cell biology, development, and cancer. Histone H3 trimethylated at lysine 27 (H3K27me3) is generated by Polycomb repressive complex 2 (PRC2) and involves a ‘reader’, EED, which recognizes a pre‐existing modified histone (H3K27me3), and a ‘writer’, methyltransferase EZH2, which modifies the histones nearby 72. Histone H3K27 methylation is removed by ‘erasers’, which prevent the maintenance/propagation of this modification. Three histone demethylases, UTX (KDM2A), UTY, and JMJD3 (KDM2B), have been reported to remove H3K27me3 73, 74. Disease‐causing mutations may affect the histone genes themselves, or the enzymes (readers, writers, and erasers) regulating the post‐translational modifications of their products.

In contrast to DNA methylation, some histone modifications and their functions are conserved from yeast to human (eg H3K4me3), but the families of enzymes catalysing the addition or removal of these modifications have expanded during evolution. For example, one single protein catalyses the deposition of H3K4me3 in yeast (Set1, part of the COMPASS complex), whereas in mammals up to six (SET1B, SET1B, MLL1, MLL2, MLL3, and MLL4) enzymes have been reported 75. This expansion follows the shift from unicellular to multicellular organisms, although the expression of each enzyme is not necessarily tissue‐specific, which explains why redundancy is often observed. PcGs, involved in the deposition of histone marks (H2AK118ub for PRC1 and H3K27me3 for PRC2) associated with transcriptional repression, were first identified in *Drosophila melanogaster* 76. The mechanism of PcG recruitment in *Drosophila* is different as these are recruited to specific DNA sequences called polycomb repressive elements 76, whereas in mammals these complexes are recruited by CpG islands 77, 78.

### Histone modification regulators in disease

*EZH2* is the most frequently mutated PRC2 component in cancer. However, both gain‐of‐function 79 and loss‐of‐function 80, 81, 82, 83 mutations have been observed in lymphoma and leukaemia, respectively (reviewed in refs 84 and 85). Certain evidence suggests that genomic loss or hypoxia‐induced down‐regulation of microRNA‐101 (miR‐101) is the cause of *EZH2* overexpression in many solid tumours 86, 87, 88, 89.

As epigenetic regulators target vast numbers of genes influencing their transcription rates, it is unsurprising that both inactivation and hyperactivation of these enzymes can lead to disease, depending on the tissue type and the developmental stage. Other genes involved in cancer have also been found to have opposing roles in different tissues. This is the case for *NOTCH1*, encoding a transmembrane receptor, which has been described as an oncogene in leukaemia 90 and a tumour suppressor gene in solid tumours 91, 92. Mutations affecting protein–protein interactions may explain these opposing effects. For example, certain missense mutations of the tumour suppressor *p53* (*TP53*) can exhibit oncogenic activities with a dominant‐negative effect achieved by the oligomerization of the mutant and the wild‐type proteins 93, 94.

Chromosomal translocations, originally identified in leukaemic cells, can also affect epigenetic regulators by creating novel fusion proteins, with different functions compared with the wild‐type protein (Figure 1 M). Almost all leukaemias and lymphomas harbour translocations (reviewed in ref 95). Chromosomal rearrangements affecting the Trithorax group *MLL* gene occur in over 70% of infant leukaemia cases 96. The resulting fusion proteins cause overexpression of a number of different target genes despite the fact that most of these rearrangements cause a deletion of the catalytic SET domain of MLL 96, 97. One mechanism of this deviant gene activation by MLL fusion proteins is the aberrant recruitment of DOT1L, a H3K79 histone methyltransferase, associated with transcriptional elongation 96, 98, 99, 100, 101.

**Figure 2:**
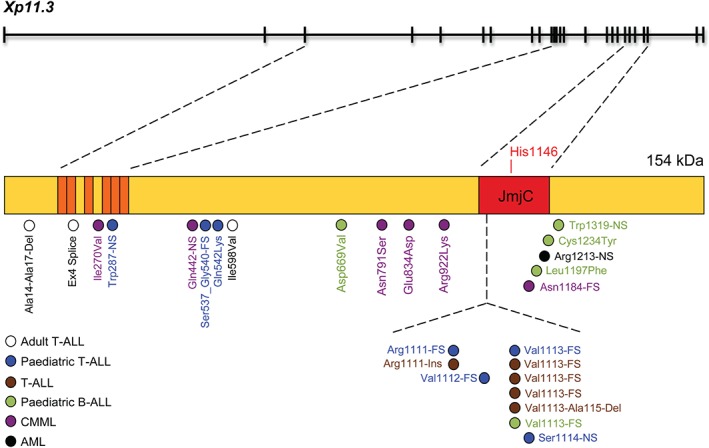
Mutations of the UTX gene in leukaemia. The UTX (ubiquitously transcribed X chromosome tetratricopeptide repeat protein) gene contains 29 exons (black boxes) that encode a 1401‐amino acid (aa) protein with a molecular weight of 154 kDa. The amino‐terminal region shows six tetratricopeptide repeat (TRP) domains (indicated in orange) and one JmjC domain (aa 1095 to 1258) which contains a catalytic histidine (His1146) (indicated in red). Blue circles depict frameshift mutations (FS) in the JmjC domain in paediatric T‐ALL 177, and white circles depict an in‐frame deletion, a splice acceptor site mutation, and a missense mutation in adult T‐ALL 104. Additional T‐ALL patients have been identified with mutations (brown circles) in the same hotspot region of the JmjC domain 120. These include three frameshift (Val1113‐FS) and two in‐frame insertions/deletions. Other mutations have been found in paediatric B‐ALL (green circles), with one frameshift, two missense, and one nonsense mutations in the JmjC domain, and an additional missense mutation between the TRP and JmjC domains 178. Other mutations have been found in CMML (purple circles) 107, 179 and AML (black circle) patients. A deletion was also detected in a patient with MDS 180. In patients with an inactivated catalytic domain, the mutant protein may have a dominant‐negative activity as the protein‐interacting TRP domain at the N‐terminus is preserved. This may allow the mutant protein to still interact with other proteins, and thus compete with the wild‐type protein (UTX for female and UTY for male) expressed by the other chromosome. Note that this gene also produces many splice variants.

Sequence conservation is high in the Jumonji C (JmjC) catalytic domain amongst the histone H3K27 demethylases, UTX (KDM2A), UTY, and JMJD3 (KDM2B) (Figure 2) 102, 103. Other domains involved in protein–protein interactions may be important for substrate specificity and segregate the function or targets of these enzymes. For example, in T‐ALL, UTX functions as a tumour suppressor, whereas JMJD3 works as an oncoprotein, despite their common enzymatic activity 104. Histone H3K27 demethylases have several functions besides their enzymatic activities, such as nucleosome depletion 105 or transcription elongation 106. Mutations seen in cancer may cause quantitative changes to overall expression, or qualitative changes that enhance or repress specific domain functions. Figure 2 depicts the *UTX* gene and its inactivating mutations found in T‐ and B‐cell acute lymphoblastic leukaemia (T‐ALL and B‐ALL), and also in chronic myelomonocytic leukaemia (CMML) 107. From this diagram it is not clear if the tumour suppressor activity of UTX depends on its demethylase activity as some mutations (affecting the TRP domain) leave the catalytic domain intact. Also, the sequence conservation within this family of enzymes makes it difficult to design specific inhibitors against each individual H3K27 demethylase (eg cross‐reactivity of GSK‐J3/GSK‐J4 for JMJD3 and UTX) 108. Moreover, epigenetic regulators do not target just histones, but other proteins also 109.

### Histone variants in disease

As described above, histones are the building blocks of nucleosomes, which are involved in chromatin packaging. Many histone variants exist, expanding the traditional roles of histones to include mechanisms such as DNA repair and maintenance of genomic stability 110. Histone H3.3 is one such variant, which is essential for mouse development, genomic stability, and normal heterochromatin function 111. The first mutations linking human disease to histone variants were identified in the genes *H3F3A* and *HIST1H3B* encoding H3.3 and canonical H3.1, respectively 112, 113. These recurrent gain‐of‐function mutations, affecting residues at or close to the position where H3K27me3 occurs, have been found in approximately 50% of paediatric high‐grade gliomas 112, 113. One of these mutations, K27M in H3.3, is a dominant‐negative inhibitor of H3K27me2/3 deposition, reducing global H3K27me2/3 on wild‐type H3.1 and H3.3 histones 114, 115, 116, 117. H3.3‐K27M prevents H3K27me2/3 deposition through direct interaction with, and inhibition of, PRC2 components 115, 116. The global reduction of H3K27me3 is concurrent with changes in DNA methylation patterns specific to tumours from H3.3‐K27M patients, leading to distinct changes in gene expression 115.

## Conclusion

To date, 125 genes with driver mutations for cancer have been discovered, and nearly half of them encode epigenetic regulators 118. The high frequency of these mutations reflects the critical role of epigenetics in disease. Disease‐specific epigenome signatures suggest that epigenetics plays an important role even in cancers where epigenetic regulators have not been mutated 49. These changes in the epigenetic landscape are strongly correlated with transcriptional changes in cancer driver genes 48. Importantly, the potency of these epigenetic regulators makes them excellent therapeutic targets; modulation of their activity by use of inhibitors could potentially reset the epigenome to a ‘normal’ state. For instance, inhibitors of DNA methyltransferases such as azacitidine (5‐azacytidine) and decitabine (5‐aza‐deoxycytidine; DAC) lead to DNA hypomethylation and have shown promising results in the treatment of MDS 119. Even when a tumour suppressor gene is missing, targeted inhibition of its antagonist can potentially reset epigenetic imbalances and mediate beneficial responses 120. To illustrate, loss‐of‐function of a H3K27 demethylase creates an imbalanced preponderance of H3K27me3 modifications in a cell. These effects could be minimized by the use of a large number of promising EZH2 inhibitors (DZNep 121, EI1 122, GSK126 123, 124, GSK926 125, GSK343 124, EPZ005687 126, CPI‐169 127, UNC1999 128, 129, and others 130). A list of current inhibitors under development for all epigenetic regulators is beyond the scope of this review, but all the current epigenetic therapies and their relevance to leukaemia can be found elsewhere 131. We must still also consider that during cancer progression, cells accumulate mutations that generate genetic and/or epigenetically distinct subclones displaying both genotypic and phenotypic heterogeneity 132. Such heterogeneity presents another challenge to treatment.

The question still remains: is a genetic mutation always required, or are pure epi‐mutations sufficient to cause a disease? Studies across many species have shown how environmental factors can directly influence phenotypes through epigenetic mechanisms. For example, queen honey bees are fed with royal jelly throughout their lifetime, with effects involving DNA methylation changes 133, gene expression changes 134, and phenotypic differences including increases in size and longevity 135, when compared with their worker bee siblings (reviewed in ref 136). Interestingly, the royal jelly contains a histone deacetylase inhibitor 137, 138 that significantly increases lifespan in *Drosophila* 135. In humans, the study of monozygotic twins (genetically identical individuals) with discordant diseases represents an excellent system with which to identify environmental causes of epi‐mutations because potential confounders (genetic factors, age, gender, maternal effects, cohort effects, etc) can be controlled 139. For example, studies of monozygotic twins showed that epigenetic differences arise during their lifetimes 140, and that twins rarely develop the same disease 141, 142. Although different somatic mutations can accumulate over time in these individuals, environmental factors causing epigenetic changes may be important in disease. A recent study on a pair of identical twins discordant for common variable immunodeficiency (CVID) revealed that differential DNA methylation was associated with deregulation of genes involved in maturation of B‐cells, but without considering potential somatic mutations that may have occurred during adult life 143. Overall, most discordant monozygotic twin studies seem to involve autoimmune, psychiatric, and neurological diseases, but also different types of cancer 139. The importance of the environment during adulthood has been shown in a recent EWAS, which has identified differentially methylated CpGs in smokers versus non‐smokers that could potentially be associated with increased breast cancer risk 53.

Epigenetic modifications also vary during lifespan and between different tissues, making disease‐causing epi‐mutations difficult to separate from normal variation. It is therefore important to ensure that age‐ and tissue‐matched reference epigenomes are available for comparison. In some cases, epi‐mutations might be inherited through the germline, suggesting a possible existence of purely epigenetically transmissible diseases. Transgenerational epigenetic inheritance studies have been described in plants 144, invertebrates 145, and mammals 146, 147, usually using changes of diet conditions as a model (reviewed in refs 148 and 149). However, these studies are mostly descriptive and require more mechanistic insights 150.

SNPs in enhancers and epi‐mutations have been strongly correlated with disease risk in many cases 51, 53, 151, 152. However, correlation does not equate to causation. Although effects of mutations in coding sequences are relatively easy to investigate, SNPs located in enhancer sequences, and associated with disease, are more difficult to validate. For example, the previously GWAS‐identified SNPs in obese patients are located in the first intron of the *FTO* gene. Smemo *et al* recently published that this intron acts as an enhancer, not for the *FTO* gene but for another gene, *IRX3*, located 500 kb away, thus revealing the role of IRX3 (and not FTO) in obesity 153. Many excellent studies mentioned in this review and beyond have aimed to dissect the mechanism by which an enhancer SNP or deletion may lead to disease, but the true ‘gold standard’ technique would be to replicate the mutation *in vivo* and examine the results. Classical gene targeting techniques have achieved this in some cases 154 but recently described genetic editing tools could make a rigorous characterization of these mutations more widely achievable 155. Similarly, the use of targeted epigenetic editing techniques 156, 157 will expand the ability of epigeneticists to investigate the phenotypes of epi‐mutations.

These recently described genome and epigenetic editing techniques could be used in the clinic to completely reset a disease‐causing mutation in a patient. Certainly, many studies are already underway investigating the use of genetic editing techniques in treating diseases such as acquired immune deficiency syndrome (AIDS) 158 and X‐linked severe combined immunodeficiency (SCID‐XI) 159 (reviewed in ref 160). Epigenetic editing, although in its infancy, is proving extremely effective and could potentially be used as a means of disease treatment 45, 157. However, particularly in cancer treatment, where mutation load can reach into the hundreds in certain tumours 118, it would be extremely difficult to correct every driver mutation in every cell. Meanwhile, the debate continues over the ethical use of genetic editing techniques as a form of disease treatment for humans 161. The use of inhibitor drugs in a clinical setting to target the effects of these mutations still remains a much more realistic option for the treatment of many cancers. As some of the proteins targeted by these drugs can have opposing effects (oncogene versus tumour suppressor) in cells from the same tissue, it is important to understand the biology of the mutations and the function of these proteins in each lineage to identify the tumourigenic pathways that they may regulate.

## Author contribution statement

AJB and DV wrote the manuscript together.
